# Implementation of physical activity on prescription for children with obesity in paediatric health care (IMPA): protocol for a feasibility and evaluation study using quantitative and qualitative methods

**DOI:** 10.1186/s40814-022-01075-3

**Published:** 2022-06-01

**Authors:** Susanne Bernhardsson, Charlotte Boman, Stefan Lundqvist, Daniel Arvidsson, Mats Börjesson, Maria E. H. Larsson, Hannah Lundh, Karin Melin, Per Nilsen, Katarina Lauruschkus

**Affiliations:** 1 Region Västra Götaland, Research, Education, Development and Innovation, Primary Health Care, Gothenburg, Sweden; 2 Unit of Physiotherapy, Department of Health and Rehabilitation, Institute of Neuroscience and Physiology, Sahlgrenska Academy, University of Gothenburg, Gothenburg, Sweden; 3 Region Västra Götaland, Centre for Physical Activity, Gothenburg, Sweden; 4 Department of Food and Nutrition and Sport Science, Faculty of Education, Center for Health and Performance, University of Gothenburg, Gothenburg, Sweden; 5 Department of Molecular and Clinical Medicine & Center for Health and Performance, Sahlgrenska Academy, University of Gothenburg, Gothenburg, Sweden; 6 Region Västra Götaland, Sahlgrenska University Hospital, Gothenburg, Sweden; 7 School of Public Health and Community Medicine, Institute of Medicine, Sahlgrenska Academy, University of Gothenburg, Gothenburg, Sweden; 8 Institute of Health and Care Sciences, Sahlgrenska Academy, University of Gothenburg, Gothenburg, Sweden; 9 Region Västra Götaland, Department of Child and Adolescent Psychiatry, Sahlgrenska University Hospital, Gothenburg, Sweden; 10 Division of Health and Society, Department of Health, Medicine and Caring Sciences, Linköping University, Linköping, Sweden; 11 Faculty of Medicine, Institution of Health Sciences, Lund University, Lund, Sweden

**Keywords:** Children, Obesity, Physical activity on prescription, Feasibility, Implementation, Determinants

## Abstract

**Background:**

Physical inactivity is a main cause of childhood obesity which tracks into adulthood obesity, making it important to address early in life. Physical activity on prescription (PAP) is an evidence-based intervention that has shown good effect on physical activity levels in adults, but has not been evaluated in children with obesity. This project aims to evaluate the prerequisites, determinants, and feasibility of implementing PAP adapted to children with obesity and to explore children’s, parents’, and healthcare providers’ experiences of PAP.

**Methods:**

In the first phase of the project, healthcare providers and managers from 26 paediatric clinics in Region Västra Götaland, Sweden, will be invited to participate in a web-based survey and a subset of this sample for a focus group study. Findings from these two data collections will form the basis for adaptation of PAP to the target group and context. In a second phase, this adapted PAP intervention will be evaluated in a clinical study in a sample of approximately 60 children with obesity (ISO-BMI > 30) between 6 and 12 years of age and one of their parents/legal guardians. Implementation process and clinical outcomes will be assessed pre- and post-intervention and at 8 and 12 months’ follow-up. Implementation outcomes are the four core constructs of the Normalization Process Theory; coherence, cognitive participation, collective action, and reflexive monitoring; and appropriateness, acceptability, and feasibility of the PAP intervention. Additional implementation process outcomes are recruitment and attrition rates, intervention fidelity, dose, and adherence. Clinical outcomes are physical activity pattern, BMI, metabolic risk factors, health-related quality of life, sleep, and self-efficacy and motivation for physical activity. Lastly, we will explore the perspectives of children and parents in semi-structured interviews. Design and analysis of the included studies are guided by the Normalization Process Theory.

**Discussion:**

This project will provide new knowledge regarding the feasibility of PAP for children with obesity and about whether and how an evidence-based intervention can be fitted and adapted to new contexts and populations. The results may inform a larger scale trial and future implementation and may enhance the role of PAP in the management of obesity in paediatric health care in Sweden.

**Trial registration:**

ClinicalTrials.gov Identifier: NCT04847271, registered 14 April 2021.

**Supplementary Information:**

The online version contains supplementary material available at 10.1186/s40814-022-01075-3.

## Contributions to the literature

- “Physical activity on prescription” is an evidence-based, complex intervention that is proven effective on adults, but neither implementation outcomes nor process has been evaluated in the context of paediatric health care.
- The hybrid-designed feasibility project combines process and outcome evaluations and uses both quantitative and qualitative methods.
- The use of Normalization Process Theory as a framework for the whole project will contribute to the literature on its application in various contexts.
- The project will contribute knowledge on the feasibility of whether and how an evidence-based intervention can be adapted and fitted to another population and context.

## Background

Childhood overweight and obesity is one of the most important global public health concerns in the twenty-first century. Prevalence has increased dramatically over the last four decades and remains high in many countries [1, 2]. The prevalence of overweight in European children aged 5–9 years was 28.9% in 2016, and the prevalence of obesity was 11.4% [3]. In Sweden, 21% of children aged 6–9 were overweight or obese in 2019, an increase by 14% since 2016 [4]. Of those 21%, 6% had obesity, up from 4% in 2016. Within this age group, the prevalence was twice as high in 9-year olds than in 6-year olds. There is also a gender difference, with an 11% obesity prevalence in boys versus 9% in girls [4]. Parent’s income level, education level, and country of birth are key predictors of lifestyle habits and obesity in children [5].

Physical inactivity is, alongside a poor diet, the main driver of childhood obesity [6]. As obesity in childhood tracks into adulthood [7, 8], addressing physical inactivity in early life is crucial. Particularly in middle childhood, social factors and physical inactivity are important risk factors for obesity [9]. Physical activity (i.e. any bodily movement that increases energy expenditure above a basal level [10]) has well-documented positive effects on physical and mental functions in children and is also important in relation to risk factors for lifestyle-related diseases, such as overweight/obesity, diabetes type 2, and to cardiovascular and metabolic risk factors [11, 12]. For children who are overweight or obese, physical activity has been shown to yield positive effects on weigh-related outcomes, especially when combined with dietary advice [13]. A recent systematic review of physical activity interventions in children with obesity showed effects on both body mass index (BMI) and physical activity [14]. Other reviews, however, are inconsistent, showing little to no effects on physical activity [15, 16].

In Sweden, national recommendations are that all children between 6 and 17 years should be physically active at least 60 min per day at a moderate-to-vigorous level [12], and that children who are insufficiently physically active should be offered physical activity counselling in health care [17]. The WHO also recommends limiting sedentary time and recreational screen time, which have shown detrimental effects on, e.g. physical fitness, metabolic health and school performance [18]. For children with obesity, physical activity at least 3 times/week at 75% of maximal heart frequency is recommended, for effects on lipids and insulin sensitivity [12]. Studies show that parents’ physical activity levels generally are linked to child physical activity levels, and parental support is perceived as crucial, indicating the benefit of involving parents in research on children [19–21].

Obesity is considered a complex multifactorial condition [22], and behaviour change interventions aiming to improve dietary intake, increase physical activity, and reduce sedentary behaviour are often prescribed and recommended [23]. One such intervention that aims to change physical activity behaviour is “physical activity on prescription” (PAP). This is an intervention developed in Sweden to promote physical activity and motivate the patient to increase their physical activity level [24]. Several studies have shown effectiveness of Swedish PAP in adult populations, including patients with overweight or obesity, measured as increased physical activity levels [25]. There is, however, a paucity of studies of PAP for children and adolescents. One small study investigated PAP in children with cerebral palsy and found PAP to be both feasible and leading to increased physical activity levels [25]. For children with obesity, no study has been identified that evaluates the implementation or effect of PAP. Before conducting a definite effectiveness trial, it is important to evaluate the feasibility and prerequisites for implementing this potentially behaviour-changing intervention in paediatric health care. The vulnerability of the patient group, the parental involvement, and the complexity of the intervention make it particularly important to investigate areas of uncertainty in a future effectiveness trial so that, e.g. recruitment, attrition, intervention fidelity, and other feasibility measures can be assessed and may be tweaked prior to a main trial.

When studying and implementing new interventions in a complex context such as health care, it is important to examine implementation determinants that can be used to develop an effective implementation strategy. Implementation determinants are factors that might prevent or enable improvements in professional practice [26] and are commonly used as an umbrella term for factors that hinder (barriers) and facilitate or enable (facilitators) implementation. They exist at several levels, including patient level, organisational level, and the wider environment/society [27]. Barriers for implementing PAP for adults that have been identified among Swedish primary healthcare providers include lack of knowledge about the intervention and lack of organisational support [28–30]. Facilitators include affirmative attitudes among colleagues and central and local supporting structures. For children, family support and parental role models are crucial when implementing physical activity, underscoring the need to involve parents in any efforts to promote physical activity [19, 31]. However, no study has investigated determinants for implementation of PAP for children with obesity.

This project addresses the above knowledge gaps. Increased knowledge and understanding of these factors are essential for successful implementation of PAP as an intervention for treatment of obesity in paediatric health care. The project will contribute important findings that can form the basis for implementation efforts in Region Västra Götaland, Sweden, and beyond. It will also provide knowledge about the feasibility of PAP for children with obesity, as well as about how an evidence-based intervention can be adapted to a new context and population and how implementation can be facilitated.

### Aims

The overarching aim of this project is to evaluate the prerequisites, determinants, and feasibility of implementing PAP for children with obesity in paediatric health care. Quantitative studies will investigate implementation and clinical outcomes, while qualitative studies will explore implementation determinants among different stakeholders.

Specific study objectives are as follows:

- Study 1: To examine how healthcare professionals and managers perceive working with PAP for children with obesity in terms of coherence, cognitive participation, collective action, and reflexive monitoring and how they perceive acceptability, appropriateness, and feasibility of a PAP intervention and, furthermore, to explore barriers and facilitators for working with PAP for children with obesity.
- Study 2: To explore experiences, barriers, and facilitators for using PAP for children with obesity among healthcare professionals and managers.
- Study 3: To evaluate the feasibility and acceptability of a PAP intervention for children with obesity on implementation outcomes and clinical outcomes, with physical activity pattern being the primary clinical outcome variable.
- Study 4: To explore children’s and parents’ experiences of participating in a PAP intervention, with a focus on perceived barriers and facilitators for implementing PAP.

Specific research questions are presented in Table 1.

**Table 1:** Research questions

*Study*	*Preliminary title*	*Research questions*
1	Working with PAP for children with obesity in paediatric health care — a cross-sectional survey	How do healthcare professionals and managers perceive working with PAP for children with obesity in terms of coherence, cognitive participation, collective action, and reflexive monitoring?
		How do healthcare professionals and managers perceive PAP for children with obesity in terms of acceptability, appropriateness, and feasibility of the intervention?
		Is there a difference in the above variables between healthcare units, age groups, or professions?
		What are the perceived barriers and facilitators for working with PAP for children with obesity?
2	Experiences among healthcare professionals and managers of using PAP for children with obesity — a focus group study	What are the experiences of healthcare professionals and managers in paediatric clinics of working with PAP for children with obesity?
		What barriers and facilitators do they perceive related to implementing PAP for children with obesity?
		Which contextual factors do they consider important when working with PAP?
3	Evaluation of PAP for children with obesity on implementation and clinical outcomes — a single-arm intervention study	Do healthcare professionals’ and managers’ perceptions of PAP and working with PAP for children with obesity change after a PAP intervention?
		Do physical activity patterns of children with obesity and one of their parents change after participation in a PAP intervention?
		Is there a correlation between the child’s and their parent’s physical activity pattern?
		Is there a change in BMI, metabolic risk markers, health-related quality of life, sleep, or self-efficacy or motivation for physical activity after participation in a PAP intervention?
		What is the acceptability and feasibility of the intervention among children and parents?
4	Children’s and parents’ experiences of participating in a PAP intervention — an interview study	What are the experiences of children with obesity and their parents of participating in a PAP intervention?
		Which barriers and facilitators of implementing PAP do children and their parents perceive?
		How do children and parents perceive their (their child’s) behaviour change, if any, with particular emphasis on physical activity behaviour?

## Methods

Findings from the individual studies will be reported according to the appropriate EQUATOR checklist [32, 33]. This protocol is reported according to the SPIRIT checklist [34], supplemented by relevant items from the CONSORT extension to pilot and feasibility trials [35]. The clinical study is registered in clinical trials (NCT04847271). Any important changes to the protocol will be amended in the registry and reported in the relevant publication. A brief structured summary of the clinical study in the format of a WHO trial registration data set is provided in Supplementary file 1.

### Study design

**Fig. 1:**
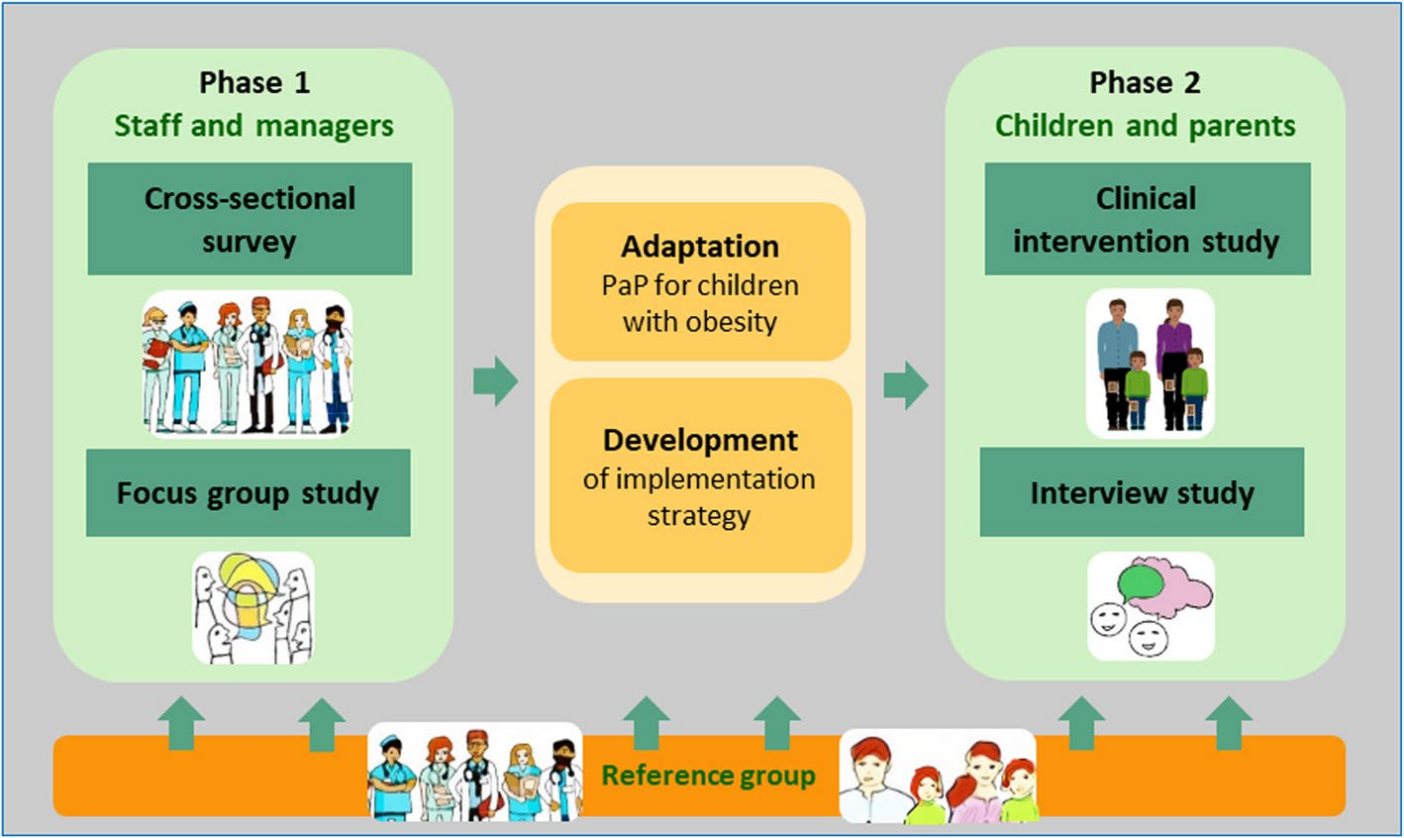
Overview of project and design of the included studies

An overview of the research project and design of the different studies is outlined in Fig. 1. The project incorporates qualitative and quantitative research methods and comprises four studies to be performed over 4 years, divided into two phases. The design, including combining feasibility with outcome evaluation, follows guidance from the Medical Research Council on assessing feasibility and evaluating complex interventions [36]. It is consistent with a hybrid type-1 approach, in which a clinical intervention will be tested while concurrently evaluating the implementation process by gathering information on its delivery and potential for implementation in a real-world situation [37]. Both implementation process outcomes and clinical outcomes will be assessed. In phase 1, two studies target healthcare professionals and managers in paediatric health care. Study 1 investigates implementation determinants among staff and managers at paediatric clinics in a cross-sectional, web-based survey. Study 2 builds on the implementation determinants identified in the survey and further explores them in a subsample of this population, using focus group discussions. Findings from these two studies will feed into an adaptation of PAP and provide input for an implementation strategy for phase 2 of the project. This second phase includes a clinical intervention in which the adapted PAP intervention is tested using a single-group before-after design (study 3) and a qualitative interview study (study 4). The clinical intervention will be evaluated using a single-group design in which children and one of their parents/guardians will take part in a PAP intervention for 4 months. Clinical outcomes in children and parents will be assessed after the intervention, with long-term follow-ups at 8 and 12 months after baseline. Pre- to post-intervention changes in implementation outcomes will also be measured using the same questionnaire as in study 1, with follow-up 12 months after baseline. Process outcome data will be collected during and after the intervention. Finally, a qualitative interview study (study 4) will explore experiences of PAP treatment among children with obesity and their parents/guardians. A reference group that includes children, parents/guardians, healthcare professionals, managers, and other stakeholders will be consulted throughout the project.

### Theoretical framework

Health care and healthcare systems have a high degree of complexity [38], involving a multitude of perspectives, structures, and stakeholders and making it a particularly challenging context in which to implement new interventions and other practices. Promoting and prescribing physical activity to children with obesity are a complex healthcare intervention, embedded by social processes surrounding the child that may involve their family, friends, the school, community, sports arenas/clubs, and the healthcare system. When studying and implementing a complex intervention in a complex context such as health care, as in this project, the use of a theoretical framework may facilitate implementation and increase the chances for its success [26, 39]. A theoretical framework for implementation that can explain social processes related to the necessary change of the childs’ behaviour is appropriate.

The Normalization Process Theory (NPT) was developed to understand and explain the social processes that frame the implementation of complex interventions in health care [38]. This theory is particularly suitable for feasibility studies of complex interventions in complex contexts, such as health care [40]. The NPT posits that implementation is a dynamic process, involving all those who carry out the implementation: healthcare professionals, organisational leaders, and patients and their families [41]. The theory is concerned with explaining what people *do* rather than their attitudes, beliefs, or perceptions and comprises 4 core constructs: coherence (sense making), cognitive participation (engagement), collective action (working/operationalising the intervention), and reflexive monitoring (evaluation). These constructs, which drive the implementation process, represent ways of thinking about implementation and focus on how interventions can become part of everyday practice [40].

In this project, the NPT was used in the planning of the project and will be used both as a framework for the qualitative studies and as a support for the clinical study in which we will examine implementation outcomes using an NPT-based instrument. Data collection, analysis, and reporting of findings will be guided by the NPT.

### Setting

The empirical arena of this research is the paediatric healthcare organisation in Region Västra Götaland, as well as the PAP clinics in Gothenburg. The paediatric healthcare organisation comprises seven specialist paediatric clinics in the city of Gothenburg, six clinics in surrounding communities, and 13 clinics in the remaining part of Region Västra Götaland. Approximately, 240 healthcare professionals (paediatric nurses, paediatricians, psychologists, dieticians, and physiotherapists) are involved in the treatment of children with obesity. For the clinical intervention, clinics in Gothenburg and surrounding communities will be invited to participate.

### The PAP intervention

The intervention that is to be adapted and evaluated in the clinical intervention (study 3) comprises PAP according to standardised procedures and, if needed, additional support provided by the PAP clinics. The PAP intervention itself consists of 3 core elements: a person-centred counselling dialogue, individually tailored physical activity recommendations with a written prescription, and individualised, structured follow-up (13). The initial dialogue is based on motivational interviewing [42], which has shown promising results in the treatment of paediatric obesity [43–45]. This counselling technique incorporates discussion about the patient’s self-efficacy beliefs and motivation for physical activity, known mediators of physical activity behaviour change [46–48].

The prescription includes recommendations for one or several types of physical activity that can be individual or group based and carried out in different settings, e.g. home, school, gym, or outdoors. Frequency, duration, and intensity of the chosen physical activity are specified. Available group activities are typically arranged by private or municipal associations.

In study 3, included children and parents/guardians will participate in an adapted PAP intervention during 4 months. The study period will be spread out over a calendar year, which will allow for seasonal variation in the activities provided and a mix of indoors and outdoors physical activity. At the end of the 4-month intervention, the participants will be encouraged to continue with their chosen activity or try another activity of their choice. To support implementation and facilitate sustainability, the intervention will be aligned with current standard care processes, existing infrastructure will be used, and the intervention will predominantly be carried out by regular staff at the paediatric clinics.

### Adaptation and implementation

Adaptation of PAP to the target group and the context will be performed in collaboration with the Centre for Physical Activity (CFFA) Gothenburg and representatives from paediatric health care, based on the findings from studies 1 and 2 and with input from the reference group. Adaptation is defined as a systematically planned and proactive process of modification aiming to fit the intervention into a new context and enhance its acceptability [47, 49]. The 2019 systematic review by Movsisyan et al. [49] will be used as guidance for the adaptation, considering the specific needs of the population and the circumstances of the paediatric healthcare organisation and assuring intervention salience and fit with the new context. The implementation determinants identified in study 1 and study 2 will be addressed in the PAP adaptation.

The identified implementation determinants also will provide input to the development of a tailored implementation strategy for those units who choose to participate in the clinical intervention. To support implementation of the adapted PAP intervention, information/education material will be developed. Relevant healthcare professionals will be offered education on the effects of physical activity, concept and components of PAP, and how to organise the structure of PAP routines on a local basis. Other educational needs identified in the survey and focus groups will also be addressed. A register of locally available physical activity service providers, e.g. sports associations, will be developed.

### Recruitment of study participants and sample size

For the assessment of implementation determinants in study 1, comprising a cross-sectional survey, all approximately 240 healthcare professionals and managers involved in the treatment of children with obesity at the different paediatric healthcare clinics in Region Västra Götaland will be invited to participate.

For study 2, comprising focus groups, we plan to recruit approximately 30 healthcare professionals of various disciplines who have experience from treating children with obesity and have some experience of using PAP. They will be recruited from paediatric clinics in Gothenburg and surrounding communities. Eligible healthcare professionals are paediatric nurses, assistant nurses, paediatricians, psychologists, dieticians, and physiotherapists. In addition, 6–10 first-line managers, senior managers, and development managers from the paediatric organisation will be recruited. A purposive sampling strategy will be used to select eligible participants.

For the assessment of clinical outcomes in study 3, we aim to recruit approximately 60 children with obesity who have been prescribed PAP and one of their parents/guardians from the paediatric clinics in Gothenburg and surrounding communities who agree to participate. Because this is a feasibility study, we have not undertaken a formal sample size calculation but aim to include a sufficient number of participants to provide information about practicalities such as recruitment and attrition rates, intervention fidelity, dose, and adherence, as well as for statistical analyses of clinical outcomes. We have also given consideration to the likelihood of a fairly large attrition rate at each time point. Uncertainties about the recruitment procedure, the capacity to achieve an appropriate sample size, and the intervention itself will be considered in terms of feasibility and whether it is of value to proceed to full-scale evaluation. Children and parents will be screened for eligibility by the staff at the participating paediatric clinics in conjunction with their first visit. All who meet the inclusion criteria will be given oral and written information about the study and asked about interest in participating. Inclusion criteria are as follows: being aged 6–12 years, diagnosed with obesity (BMI > ISO-BMI 30), having an insufficient physical activity level according to national recommendations, and having a parent who is willing to participate. Insufficient physical activity level is defined as not reaching the national guidelines recommendation of 60 min of moderate to vigorous physical activity per day [12]. Exclusion criteria are as follows severe psychiatric comorbidity, severe intellectual or physical disability, or planning to relocate outside the study area within 12 months. To support recruitment and reach the desired sample size, site coordinators will be appointed, trained, and provided with adequate study material.

For study 4, we expect to recruit approximately 10–15 children-parent pairs. Sample size cannot be determined in advance because data collection should ideally go on until no new information emerges in the interviews. Eligible children and parents/guardians will be identified in collaboration with staff at the involved clinics. A purposive sampling strategy will be used, aiming for maximum variation in gender, age, parent’s gender, age, and education. Inclusion criteria will include being aged 6–12 years, diagnosed with obesity, having an insufficient physical activity level, and having a parent who is willing to participate. Parents will be approached and invited to participate by the project coordinator, who will provide oral and written information about the study and its aims and give the opportunity to ask questions.

### Patient involvement

Both users (healthcare professionals) and end users (parents of children with obesity) have been involved in the design and planning of the project. We will continue to solicit user input throughout the project. We are collaborating with the patient organisation HOBS (health independent of size). User representatives are included in the reference group. The reference group will be consulted on an as-needed basis (e.g. in the recruiting process, the adaptation of PAP, the development of the clinical intervention, and in developing intervention material). Children’s and parents’ views and experiences of PAP will be solicited in interviews (study 4), which will contribute to evaluation of the PAP intervention and its feasibility, and provide input to further development of the intervention.

### Data collection and outcomes

#### Implementation outcomes

Implementation outcomes will be measured pre-implementation, post-intervention, and at 12-months follow-up. Data will primarily be collected from healthcare professionals and managers through a web-based questionnaire comprising several validated instruments. Primary implementation outcomes are the four core constructs of the NPT: coherence, cognitive participation, collective action, and reflexive monitoring. They will be assessed using the Normalization MeAsure Development (NoMAD), which measures implementation from an NPT perspective and reflects the four constructs [50, 51]. The validated Swedish version S-NoMAD [52] is used in this project and has been adapted to fit children with obesity and address both healthcare professionals and managers. The S-NoMAD comprises 23 items, answered on 11-point or 5-point Likert-type scales.

Secondary implementation outcomes are the acceptability, appropriateness, and feasibility of implementing PAP in paediatric health care. Acceptability is defined as the perception among stakeholders that a given treatment, service, practice, or innovation is agreeable, palatable, or satisfactory; appropriateness is the perceived fit, relevance, or compatibility of the innovation or evidence-based practice for a given practice setting, provider, or consumer and/or perceived fit of the innovation to address a particular issue or problem; and feasibility is the extent to which a new treatment, or an innovation, can be successfully used or carried out within a given agency or setting [53]. These outcomes will be measured with the Acceptability of Intervention Measure (AIM), Intervention Appropriateness Measure (IAM), and Feasibility of Intervention Measure (FIM) [54], all validated instruments with the purpose of assessing the fit and match of a practice or intervention to a given context, targeting different criteria [54]. The three measures comprise 4 items each, answered on 5-point Likert-type scales. Before use, they were translated and cross-culturally adapted into Swedish and adapted to children with obesity, following established procedures including forward and backward translation and validating the translation using cognitive debriefing [55, 56]. The translation aimed to produce a language version with conceptual equivalence with the original and relevance to the new target culture and context.

Barriers and facilitators will be explored in two open-ended questions, in which the respondents will be given the opportunity to describe their experiences and thoughts regarding determinants for implementing PAP in their clinic, expressed in their own words. They will also be further explored in the focus group study.

Acceptability of the intervention from the child’s perspective will be measured with the Client Satisfaction Questionnaire (CSQ-8) [57], translated to Swedish and adapted to children and parents. Feasibility for the child to participate in PAP will also be examined by collecting process data on fees, equipment, and transport costs of the chosen physical activity, as well as travel time, time spent at the activity, and at assessments and other contacts during the intervention.

Additional implementation process outcomes will be assessed as follows: recruitment and attrition rates will be examined. When possible and if participants consent, reasons for dropout will be documented. Intervention fidelity (intervention delivery as intended) and dose (quantity of intervention) will be captured by monitoring intervention components delivered. Other simultaneous treatment, e.g. dietary recommendations, will be documented. Availability of local physical activities will be documented. Data regarding intervention adherence and participation in the chosen physical activities will be collected via analogue and/or digital activity diaries.

#### Clinical outcomes

All clinical outcomes will be measured pre- and post-intervention and at 8 and 12 months’ after baseline. The primary clinical outcome will be physical activity level, measured as minutes per day spent in moderate to vigorous physical activity (MVPA) and assessed using the Axivity accelerometer (Axivity AX3, Axivity Ltd., UK). Accelometry is the most commonly used method in clinical and epidemiological research for objective measurement of physical activity, is easy to use for the study participant and the researcher, and has a high sensitivity for detecting changes in physical activity (30). The accelerometer will be placed above the child’s and parent’s hip in an elastic belt and worn 24 h per day for seven consecutive days. In this position, the accelerometer can be used to assess physical activity at different intensities. The accelerometer recording is accompanied by an activity diary to determine bedtime, non-wear time, and sport activities. A strict protocol for data quality assurance (malfunction, spurious data, wear time, valid days) will be used. A project coordinator will provide the accelerometer to the participants together with oral and written instructions, support the participants if necessary, and collect the accelerometer after use. Measurement procedure, quality assurance, data processing, and analysis will be supported by an experienced team at the Center for Health and Performance [58].

Secondary clinical outcomes will be physical activity patterns (time spent in different types of activities and at different intensity levels, including sedentary time) measured with accelerometry; anthropometric measures, i.e. BMI; and waist circumference, collected from medical charts. Metabolic risk markers will be assessed via blood pressure measures and blood samples, including fasting plasma glucose, high- and low-density lipoprotein cholesterol, insulin resistance, and triglycerides. In addition, patient-reported health-related quality of life will be measured using KIDSCREEN-10 [59], and the child’s sleep will be measured using the Insomnia Severity Index (ISI) [60, 61]. Parents’ perception about their child’s sleep will be measured using the Pediatric Insomnia Severity Index (PISI) [62, 63]. The child’s self-efficacy for physical activity, and motivation for physical activity, will be measured with validated instruments adapted to children with obesity and parents and appropriately translated to Swedish [64–67]. Adverse events will be monitored and documented.

Demographic data on children will be collected at baseline, including age, gender, comorbidities, and country of birth. Data on participating parents/guardians include age, gender, BMI, comorbidities, country of birth, and education level.

To enable missing data analysis, demographic data will also be collected for those who decline participation, when possible.

#### Qualitative data

For study 2, data will be collected from healthcare professionals and managers using focus group methodology [68]. Semi-structured focus group discussions will be conducted, with groups comprising a mix of professions. Managers will form separate focus groups. The focus group methodology was chosen to stimulate discussion among the participants and capture collective interaction and experiences. The discussions will be facilitated by a moderator and an observer. Discussions will be recorded and transcribed verbatim. A discussion guide will be developed to ensure that adequate data responding to the research questions are collected, and that the four NPT constructs are covered in the discussions.

For study 4, data will be collected in individual, semi-structured interviews with children who have participated in the PAP intervention and one of their parents/guardians. A purposive sampling strategy will be used, aiming to ensure maximum variation in the sample (e.g. child gender and age, parent gender, age, education level, and country of birth). Data collection will continue until no new information seems to be forthcoming in the interviews. The interviews will be recorded and transcribed verbatim. An interview guide will be developed to ensure that adequate data responding to the research questions are collected.

### Data analysis

#### Implementation outcomes

Implementation outcome data will be analysed in two steps. First (in study 1, where the purpose of this survey is to assess healthcare providers’ perceptions of PAP treatment with the aim of identifying implementation barriers and facilitators), baseline (pre-intervention) data will be analysed descriptively using absolute and relative frequencies, as well as means and standard deviations. Differences between age groups, years’ of experience, and different professions will be analysed using chi-square tests We will do a subgroup analysis of respondents from the Gothenburg clinics in which PAP has already been implemented, including access to extra support from the PAP clinics. Second (in study 3), in the subset of the study 1 participants in which the intervention is carried out, we will perform longitudinal analysis of changes after participating in the intervention, using data from pre- and post-intervention, and follow-up at 12 months after baseline. Average change over time will be analysed across the three time points using mixed linear modelling, given the assumption that data are normally distributed. Analyses will be per protocol, and missing data will be handled with maximum likelihood estimation. The free text answers to the open-ended questions related to implementation determinants will be analysed using qualitative content analysis [69].

#### Clinical outcomes

Descriptive statistics will be used to summarise characteristics of the participants and will be presented as means with standard deviations or frequencies and percentages. For physical activity outcomes, raw accelerometer data will be processed and filtered in several steps to a measure of activity intensity (mg) [58]. Previously developed and tested calibration equations for body positions, activity type, and for activity intensities will be applied [70, 71]. Accelerometer data will be analysed in 3-s epochs in order to accurately detect normal variation in physical activity [58]. Continuous data on physical activity patterns at baseline, 4, 8, and 12 months, will be analysed using multivariate regression analysis for repeated measures, controlled for multicollinearity between intensity levels. To examine potential influences of demographic and parental factors (gender, age, education level, country of birth) on physical activity patterns, the models will be adjusted for these variables. Change in absolute BMI in kg/m^2^ and in metabolic risk markers in mmol/L will be calculated and analysed using mixed linear modelling. Standardised measures for the separate risk factors waist circumference, insulin resistance, systolic and diastolic blood pressure, high-density lipoprotein, and triglycerides will also be combined in a so-called MetS score and analysed using mixed linear modelling. Patient-reported outcomes will also be analysed using mixed linear modelling and adjusted for the same demographic and parental factors. Changes over time will be presented with 95% confidence intervals. The self-efficacy and motivation variables’ potential mediating role for physical activity will also be explored by including these parameters into the models as covariates. Statistical significance will be set at *p* < 0.05.

#### Qualitative data

Data from the focus group discussions will be analysed using qualitative content analysis for focus groups [68]. A combination of inductive and deductive analysis will be used. Codes will first be developed inductively from the transcribed focus group sessions, and then, in a deductive approach using NPT as a coding framework, sorted into categories that reflect the four constructs of the NPT. Findings that cut across categories will be developed into one or several themes capturing the essence of the discussions and allowing for interpretation and a higher level of abstraction.

Data from the interviews with children and their parents will be analysed inductively using qualitative content analysis reflecting manifest and latent content of the interviews [69]. Meaning units of the transcribed interviews will be identified, condensed, and coded. Codes will be compared and, based on similarities, classified into categories and subcategories that describe the manifest content. Lastly, one or several overarching themes will be formulated based on the latent content that can be discerned across the categories.

### Ethical considerations and data management

In study 1, written participant information is provided via a link in the email that invites participants to answer the survey, and participants acknowledge having read the information and provide their consent by checking a box in the survey. In studies 2–4, written informed consent will be obtained from all participants. In studies 3–4, children and their parents/guardians will be provided oral and written information about the respective study, and informed parental consent and child assent will be obtained by the project coordinator or a site coordinator. Written information will be developed in a child version and a parent version. Because we consider it important to not exclude eligible non-Swedish-speaking children and parents, several language versions will also be developed, and interpreters will be available for those who do not speak Swedish or English.

A data management plan (DMP) has been developed, providing details on how data will be collected and managed throughout the project, as well as how they will be documented, stored, and archived after the project has been concluded. The DMP also contains information about how, when, where, and to whom data will be made available. The DMP can be provided on reasonable request from the principal investigator. Routines for data storage from Region Västra Götaland will be followed, and metadata standards will be used to describe the data material. A data management team will manage and monitor data accuracy and quality and comprises the principal investigator, the project coordinator/doctoral student, a postdoc researcher, and a research assistant. All study data, including the final study dataset, will be treated confidentially, and access will be restricted to the research team. When reporting findings, no data will be traceable to individual participants.

### Timeline

A timeline and schedule of enrolment, intervention, and assessments is presented in Table 2.

**Table 2:**
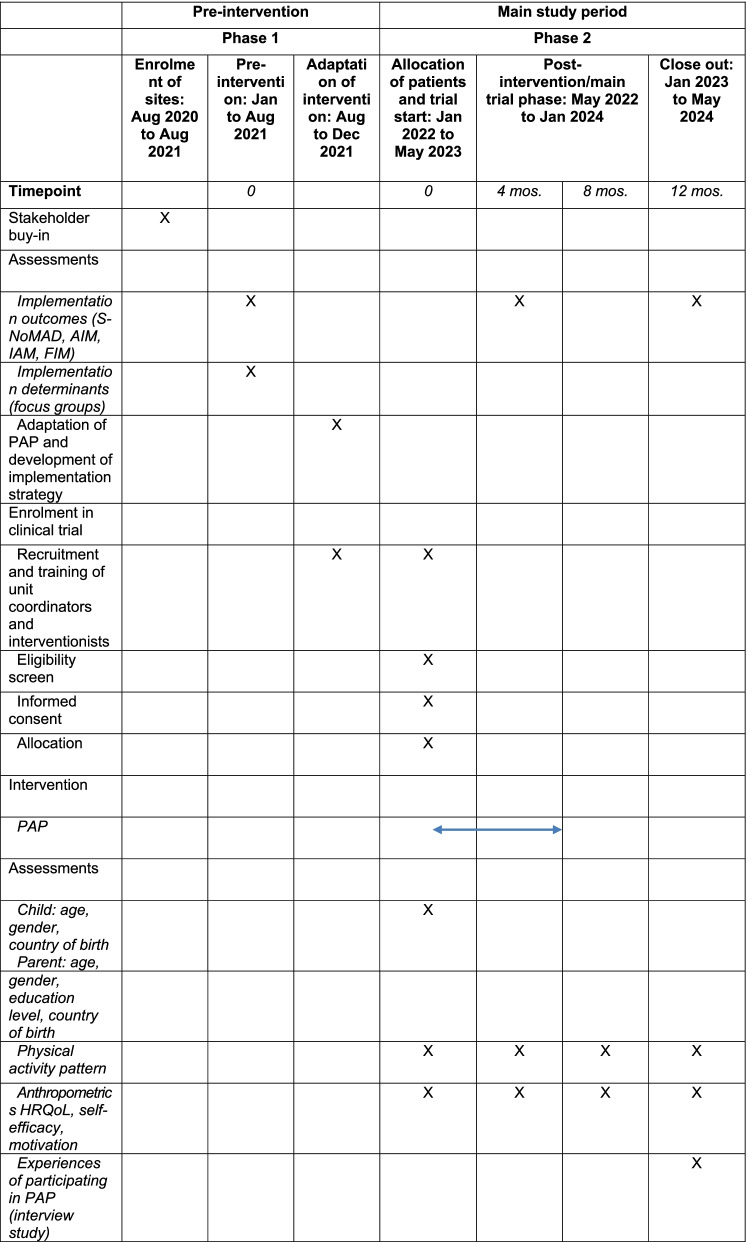
Timeline of enrolment, intervention, and assessments

### Dissemination and implementation of study findings

The study findings will be reported in peer-reviewed scientific journals and social media and presented at seminars and national and international conferences. The results will also be disseminated through regular dissemination and communication channels, within and outside health care and university contexts. Summaries of results will be provided to the participants.

## Discussion

This research project investigates the feasibility of implementing physical activity on prescription to address childhood obesity, a global health issue of great and growing importance. The pre-implementation, hybrid design feasibility project was designed to evaluate the prerequisites, determinants, and feasibility of implementing PAP adapted to children with obesity and to explore the experiences of children, parents, and healthcare providers related to their experiences, attitudes, and perceived determinants for implementation. Implementation outcomes will be collected using questionnaires, based on validated instruments, before, during, and after a pilot implementation in participating paediatric clinics. Clinical outcomes will be collected from children and parents pre- and post-intervention and at 8 and 12 months from baseline. The process will be followed closely, and process evaluation data will be collected during and after the intervention. The project is expected to show whether PAP is a feasible intervention and has a place among other treatment options in paediatric health care.

The hybrid type-1 implementation-effectiveness approach is a useful design that allows parallel evaluation of the implementation process, preconditions, and determinants, as well as clinical outcomes [37]. It is particularly suitable in a context where the intervention is supported by evidence in one population but needs to be adapted and tested in another population while simultaneously assessing barriers and facilitators to “real-world” implementation of this intervention. This design is often used when testing an intervention in a randomised controlled trial design but can also be applied for the type of single-arm feasibility study in which an intervention is evaluated using a before-after approach. An important advantage of the hybrid design is that it could speed up the process of translating research findings into routine practice and allow for addressing many implementation research questions earlier than what could be achieved in a more traditional, linear “efficacy-effectiveness-implementation” approach [37]. The combination of outcome and process evaluation is particularly relevant in interventions that involve multiple stakeholders and, as in this case, partially is conducted in community settings where contextual factors will influence intervention outcomes [72].

Our choice of NPT as a theoretical framework for the project was based on its focus on the social processes surrounding the child, involving multiple stakeholders both within and outside the healthcare system. In addition to guiding implementation planning, its use also may help explain social processes related to implementing PAP in paediatric health care. The PAP intervention is complex and involves multiple healthcare professions and a range of other stakeholders, in addition to the child itself and their family. It also includes multiple processes that may be better understood through the lens of an implementation theory such as the NPT. Further advantages of using NPT is its empirical grounding in health care and its description as being stabile, robust, and user-friendly [73].

There is an abundance of measures to choose from when assessing implementation outcomes, with a varying range of psychometric quality [74]. We strived to select the most appropriate, relevant, and validated measures. The NPT-based S-NoMAD will be used to assess the implementation process, with a focus on the collective action employed in the different work processes involved in the treatment of children with obesity in the paediatric healthcare context. We chose the NPT constructs as primary implementation outcomes because they represent the mechanisms that shape the implementation process looking into a real-world setting, in this case a paediatric healthcare organisation. Furthermore, the S-NoMAD can be used to point out problems that can be addressed when implementing the intervention, as well as to assess the implementation process over time [37]. Supplementing S-NoMAD with the AIM, IAM, and FIM instruments will allow us to also assess implementation outcomes that are important determinants for implementation success. These outcomes have been proposed to be the most salient implementation outcomes to measure before the start of an implementation process in which an evidence-based practice is adapted and implemented [53]. The instruments are very brief, giving excellent usability, and have been rigorously developed and psychometrically tested [54].

A challenge when adapting a clinical intervention such as PAP that has been evaluated and found effective in another context is that adaptation by default means that intervention fidelity is not maintained [49]. Fidelity is considered important for implementation success [75] and an important part of a process evaluation [76]. However, a high degree of intervention fidelity requires strict adherence to a protocol and implies a top-down approach to implementation, whereas adaptation that involves engaging users and other stakeholders reflects a bottom-up approach [77]. This approach is not only more politically appealing from a social development perspective but is also likely to improve acceptability and feasibility of the intervention in the new context.

We chose physical activity level as primary clinical outcome because increasing physical activity is the main goal of the PAP intervention and because the effect of PAP on activity level has been established in adults [78]. We will assess physical activity objectively using accelerometers in this project as it reduces measurement errors associated with subjective methods, especially in children [58]. In addition, a robust measurement protocol will be applied to assure a high-quality standard, considering all steps from data collection to processing to achieve useful and reliable measures [58].

We will collect clinical outcome data immediately post-intervention and again at 8 and 12 months from baseline. It is well known that behaviour change takes time, and continued physical activity will be encouraged at the end of the intervention period. Conversely, it has also been shown that a positive change post-intervention, for example in BMI, may not be sustained in the long term [13].

PAP has already been used at several paediatric healthcare units, to varied extents, both in Region Västra Götaland and elsewhere in Sweden. This, together with the fact that PAP is a well-established intervention for adults, constitutes a clinical advantage and should facilitate a wider implementation of the intervention, if found effective, throughout the region and, later, to other regions in Sweden and potentially elsewhere. This project is expected to generate new knowledge regarding the feasibility of adapting PAP to children, as well as about whether and how an evidence-based intervention can be fitted and adapted to new contexts and populations. The qualitative study findings will increase our understanding of implementation determinants, including contextual factors, and of childrens’ and parents’ experience of participating in a PAP intervention. The study results may form the basis for a larger scale clinical trial, may guide future implementation, and may potentially enhance the role of physical activity in the management of obesity in paediatric health care in Sweden. If the adapted PAP intervention shows positive results on physical activity levels and other outcomes for the children included in this study, the prospects are good for implementing the intervention in routine paediatric health care.

### Strengths and limitations

Main strengths of the project are the hybrid design with assessment of both implementation and clinical outcomes, the use of both quantitative and qualitative methods and validated outcome measures, and the objective measurement of the primary clinical outcome. Further strengths include the application of a well-established, appropriate implementation theory in both quantitative and qualitative studies, the involvement of children and parents throughout the project, and the long-term follow-up. The main limitation of the project is the lack of control group and randomisation, precluding conclusions about effectiveness of the adapted PAP intervention. Another limiting factor is the small sample size in study 3, which may make the study underpowered and limit generalisability of the results to a larger population.

## Supplementary Information

**Additional file 1: Supplementary file 1**: WHO trial registration data set.

**Additional file 2: Supplementary material**: SPIRIT 2013 Checklist: Recommended items to address in a clinical trial protocol and related documents.

## Data Availability

The complete study protocol, anonymised participant level dataset, and statistical code for generating the results will be available on reasonable request from the principal investigator, after the study results have been published.

## Abbreviations

AIM: Acceptability of Intervention Measure
BMI: Body mass index
CFFA: Centre for Physical Activity Gothenburg
DMP: Data management plan
FIM: Feasibility of Intervention Measure
IAM: Intervention Appropriateness Measure
NoMAD: Normalization Measure Development
NPT: Normalization Process Theory
PAP: Physical activity on prescription
