# Supplementary material for: Implementation of physical activity on prescription for children with obesity in paediatric health care (IMPA): protocol for a feasibility and evaluation study using quantitative and qualitative methods
Source: Pilot Feasibility Stud. 2022 Jun 1;8:117. doi: 10.1186/s40814-022-01075-3 (PMC9158137; doi:10.1186/s40814-022-01075-3)
Supplement: Supplementary file 1 — Additional file 1: Supplementary file 1: WHO trial registration data set. [file 40814_2022_1075_MOESM1_ESM.docx]

**Supplementary file 1** WHO trial registration data set

| **Data category** | **Information** |
| --- | --- |
| Primary registry and trial identifying number | ClinicalTrials.gov NCT04847271 |
| Date of registration in primary registry | 14 April, 2021 |
| Secondary identifying numbers | Not applicable |
| Source of monetary or material support | FORTE – the Swedish research council for health, working life and welfare, grant no. 2020-01244 |
| Primary sponsor | FORTE – the Swedish research council for health, working life and welfare |
| Secondary sponsor(s) | Not applicable |
| Contact for public queries | Dr. Susanne Bernhardsson, email susanne.bernhardsson@vgregion.se |
| Contact for scientific queries | Dr. Susanne Bernhardsson, email susanne.bernhardsson@vgregion.se |
| Public title | Implementation of physical activity on prescription for children with obesity (IMPA) |
| Scientific title | Implementation of physical activity on prescription for children with obesity in paediatric health care (IMPA): a feasibility study |
| Countries of recruitment | Sweden |
| Health condition studied | Obesity |
| Intervention(s) | Intervention: Physical activity on prescription (PAP), including the following three core components: 1) a person-centred dialogue, 2) a written prescription with individualised recommendations for physical activity, including type of activity, frequency, dose, and 3) a structured follow-up. Comparator: None, this is a single-arm study |
| Key inclusion and exclusion criteria | Ages eligible for study: 6-12 years; Sexes eligible for study: both; Accepts healthy volunteers: no  Inclusion criteria: children (6-12 years old) with obesity (BMI>ISO-BMI 30), insufficient physical activity level, a parent willing to participate.  Exclusion criteria: severe psychiatric comorbidity, severe intellectual or physical disability, planning to relocate outside study area within 12 months |
| Study type | Interventional |
| Date of first enrolment | November 2021 |
| Target sample size | 60 |
| Recruitment status | Not started |
| Primary outcome(s) | Physical activity level |
| Key secondary outcomes | Physical activity pattern, including sedentary time and time in different types of activities at different intensity levels; anthropometric measures (BMI and waist circumference); patient-reported health-related quality of life, self-efficacy for physical activity and motivation for physical activity |
